# An Unusual Presentation of a Black Discoloration on the Tympanic Membrane of a 10-Year-Old Girl: A Case Report

**DOI:** 10.7759/cureus.66564

**Published:** 2024-08-10

**Authors:** Ethan Dimock, Rafi Haddad, Rhiannon Boudeleh, Alise Haddad, Michael Haupert

**Affiliations:** 1 Otolaryngology, Oakland University William Beaumont School of Medicine, Auburn Hills, USA; 2 Otolaryngology, University of Balamand, Balamand, LBN; 3 Medicine, University of Balamand, Balamand, LBN; 4 Otolaryngology, Corewell Health William Beaumont University Hospital, Royal Oak, USA

**Keywords:** middle ear disorders, middle ear injuries, tympanic membrane discoloration, paediatric otolaryngology, general otolaryngology

## Abstract

This case report details an unusual presentation of unilateral tympanic membrane discoloration in a 10-year-old girl. The mysterious black discoloration was explored by various medical specialties, revealing a complex diagnostic journey due to the lack of evidence for this specific finding. Initially, the patient consulted her primary care physician after inserting a graphite pencil into her left ear canal, but without associated symptoms, she was considered to have returned to her baseline. The abnormal discoloration on the left tympanic membrane was first observed 10 months later, following diagnoses of two episodes of otitis media, otitis externa, and a middle ear effusion over three separate visits. By this time, the patient had been seen by four different medical professionals. The lesion was described as “a blackish discoloration in the posterior superior quadrant of the unperforated tympanic membrane near the umbo.” This report underscores the need for thorough evaluation and consideration of atypical presentations when encountering unusual tympanic membrane discolorations.

## Introduction

In pediatric otolaryngology, ear foreign bodies are relatively common and typically present initially at emergency centers. These incidents often involve objects inserted by the child into their ear [1]. Studies show that such cases most frequently occur in children under five years old, with a male-to-female ratio of 1.33:1. They usually present within 24 hours of the incident, originate in the right ear, and are generally uncomplicated [1]. Common foreign bodies in this age group include food, plastic toys, and small household items [2]. When the inciting event is not observed, nonspecific symptoms can lead to delayed or incorrect diagnoses [2]. Complications may arise from the object itself, trauma during removal, or if the object remains undetected for an extended period of time. Although most cases are resolved without major issues, potential problems include abrasions, bleeding, and tympanic membrane perforation [1]. Therefore, it is crucial for both ENT and primary care physicians to maintain a high index of suspicion for unexplained upper airway symptoms, especially in this age group.

## Case presentation

This case describes the clinical course of a 10-year-old Caucasian female patient who ultimately presented with an unusual black discoloration on the left tympanic membrane at an otolaryngology clinic in a hospital outside Detroit, Michigan, United States, after a circuitous diagnostic journey. The sequence of events began over a year before she visited this specialist.

Her initial encounter was with her pediatrician, who practiced at a private office near Detroit, Michigan. The pediatrician noted that the patient had inserted a pencil into her left ear three days prior and was experiencing pain from this trauma. The examination revealed a mildly erythematous left ear canal but showed no signs of infection or trauma. Similarly, the tympanic membrane appeared normal, with no signs of perforation. The initial treatment plan was to monitor for redness, swelling, or increasing pain and to return to the pediatric office within 48-72 hours if there were no signs of improvement.

Due to the persistence of her symptoms, the patient returned a few days later to the same pediatric group. Although she visited the same medical center, she was seen by a different physician within the group. This physician diagnosed her with nonrecurrent acute suppurative otitis media of the left ear. Notably, the tympanic membrane, which had been described as normal during the first visit, was now reported to have spontaneously ruptured. It is unclear whether this physician was aware of the previous trauma, as it was not mentioned in the chart. The patient was prescribed oral amoxicillin-clavulanate at 45 mg/kg/day, to be taken every 12 hours for 10 days. She was instructed to avoid water in the ear and to monitor the ear drainage and pain. Despite this treatment, the infection did not subside, and the perforated tympanic membrane remained unhealed after two weeks. The antibiotic was then changed to oral cefdinir at 14 mg/kg/day for 10 days. Nearly three weeks later, a different provider at the same medical center confirmed that the otitis media had resolved.

Five months after resolving her previous symptoms, the patient returned to the pediatric clinic with complaints of left ear pain, along with a cough, congestion, and purulent drainage. The fourth provider she saw at the medical center diagnosed her with non-recurrent acute suppurative otitis media of the left ear and noted a spontaneous rupture of the tympanic membrane. She was prescribed oral amoxicillin (400 mg/5 mL in a reconstituted suspension and 12.5 mL every 12 hours for seven days) and ofloxacin (five drops in the left ear twice daily for seven days).

She showed significant improvement until four months later, when she presented again with symptoms of bilateral pain and discomfort. During this visit, the same pediatrician, who had previously noted the spontaneous rupture of the tympanic membrane, diagnosed her with bilateral middle ear effusion and prescribed fluticasone (one spray into both nostrils once daily). Additional diagnoses included allergic rhinitis and acute otitis externa of the left ear. Ofloxacin (four drops into the left ear twice daily for seven days) was prescribed for the acute otitis externa, and the infection was resolved by a follow-up visit three weeks later.

For the first time, the physician observed a black mark on the left tympanic membrane, described as “a black streak seeming to overlay her left tympanic membrane.” The rest of the external auditory canals were unremarkable bilaterally, and the nose and oropharynx were clear and without sores, with moist mucous membranes. Due to the mysterious nature of the tympanic membrane finding, the patient was referred to an otolaryngology clinic for further evaluation.

During her visit, the patient informed the otolaryngologist of persistent aural fullness lasting several months. The black discoloration was observed in the same location noted previously, prompting the ENT physician to recommend further evaluation. Both the tympanogram and audiogram results were normal bilaterally, leading to a referral for a CT scan to rule out potential malignancies, such as melanoma, of the tympanic membrane.

**Figure 1:**
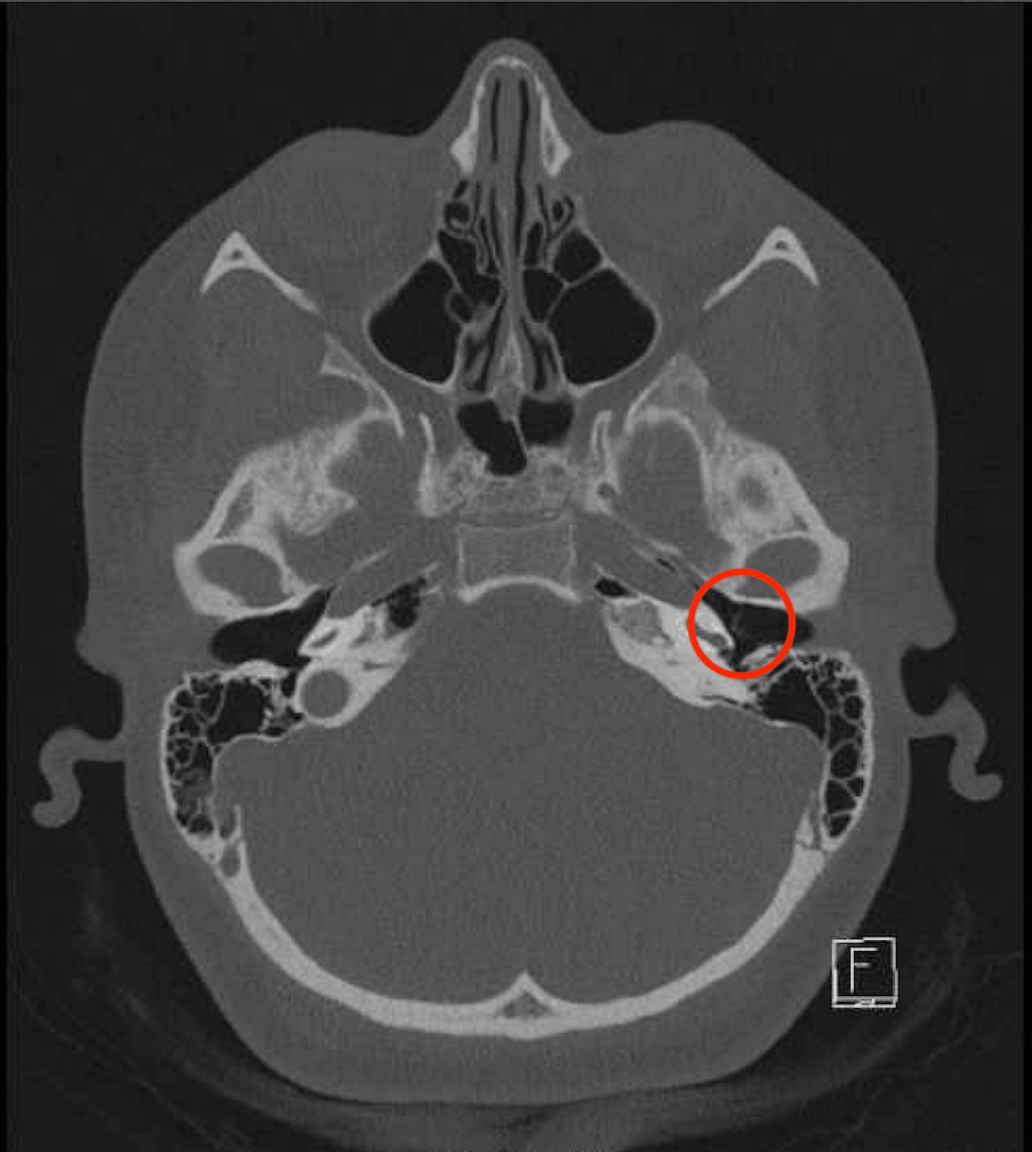
Axial view of a temporal CT scan without contrast, showing a thickened left tympanic membrane (highlighted with a red circle)

**Figure 2:**
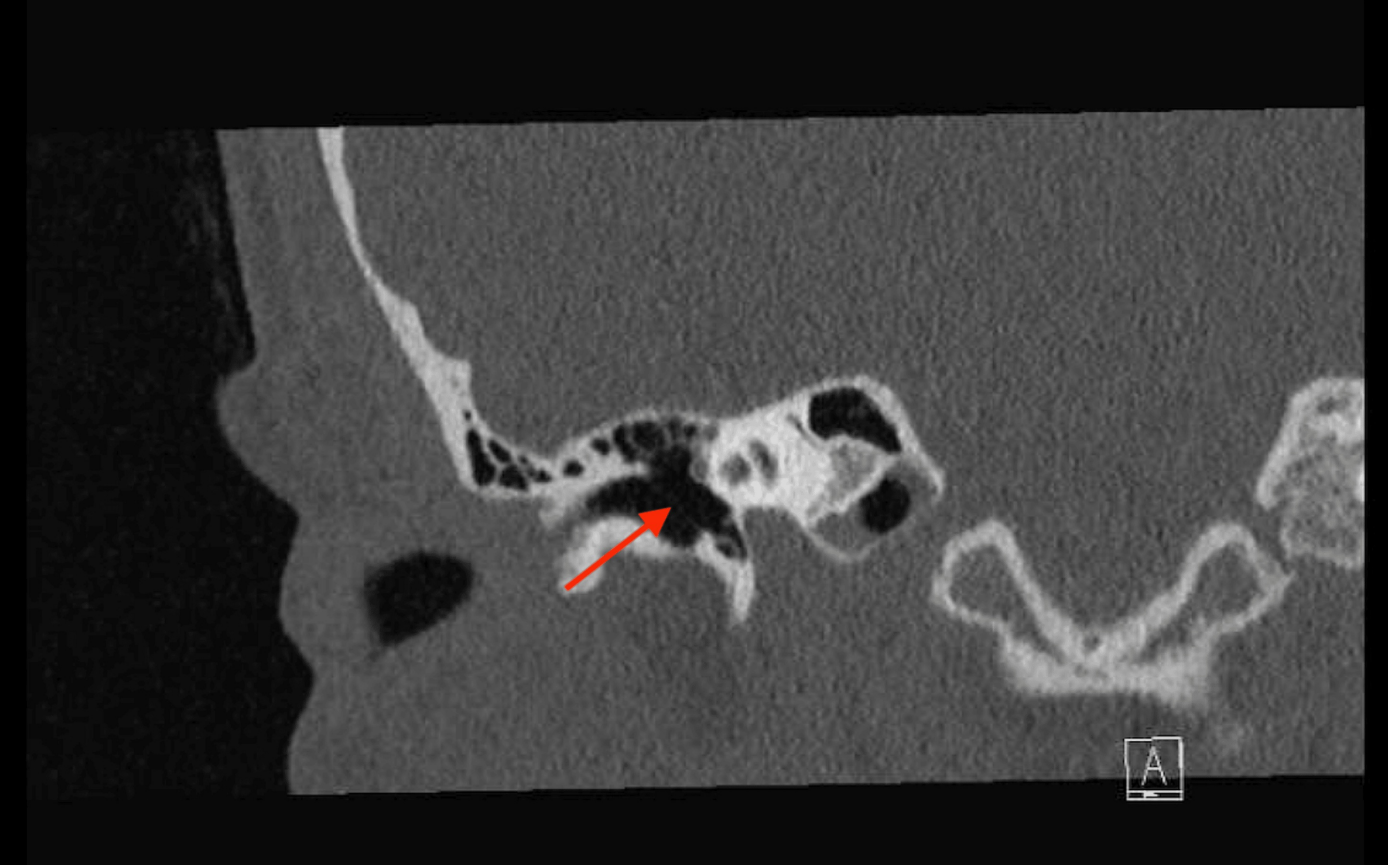
Coronal view of a temporal CT scan without contrast, showing the right internal acoustic canal with a normal right tympanic membrane (indicated by a red arrow)

**Figure 3:**
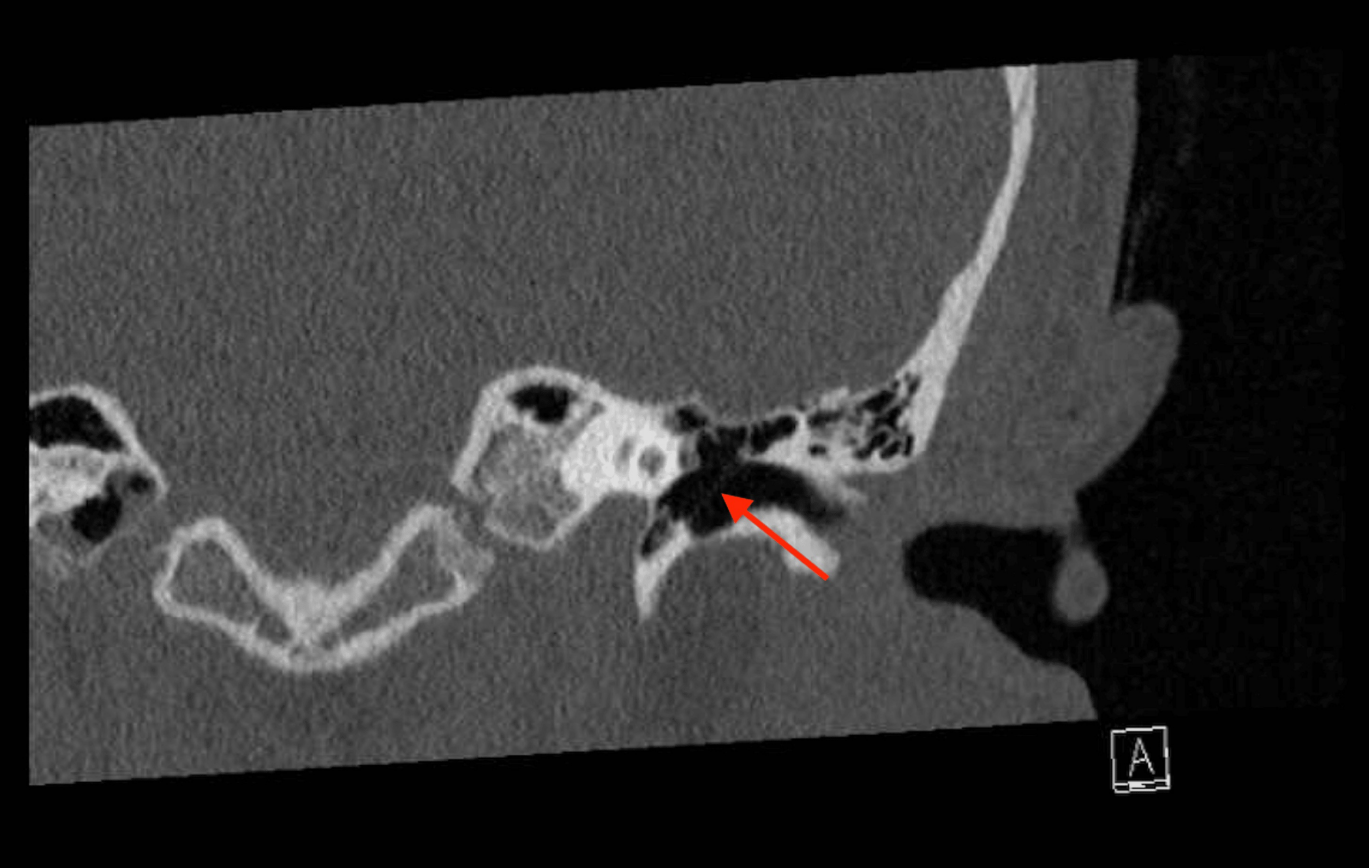
Coronal view of a temporal CT scan without contrast, displaying the left internal acoustic canal with a thickened left tympanic membrane (indicated by a red arrow)

The CT scan revealed only a thickening of the left tympanic membrane compared to the right (Figures 1, 2, 3), which was not indicative of malignancy. The abnormal thickening raised suspicion of a “tattooing effect” on the eardrum, resulting from the trauma of the pencil insertion. This effect involves scarring and residual pigment from the traumatic event. Contrary to previous assessments, the otolaryngologist found no evidence of perforation or granulation tissue and observed an entirely intact tympanic membrane.

**Figure 4:**
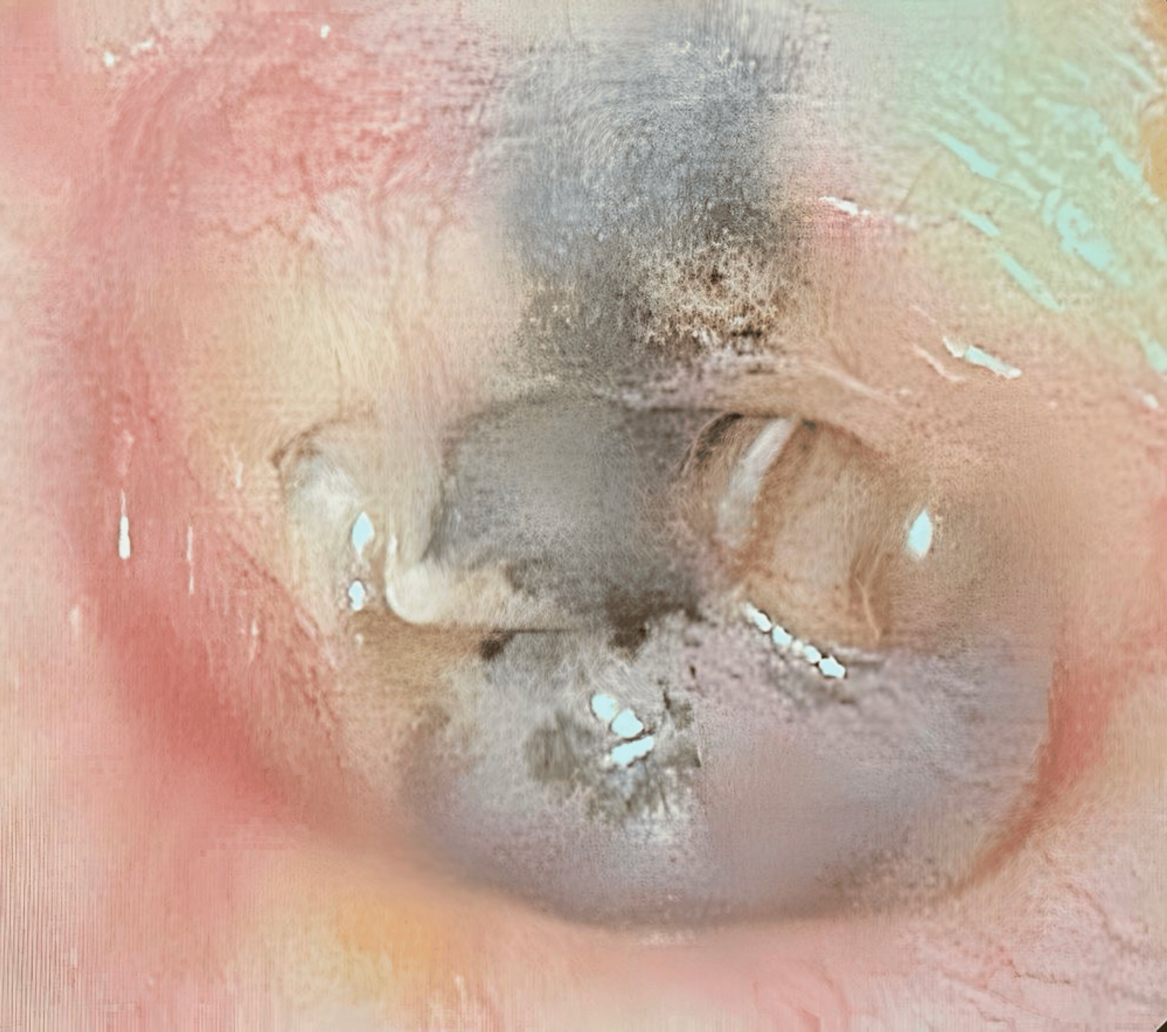
Black pigmentation on the left tympanic membrane

Eight months later, the patient returned to the pediatric otolaryngologist for follow-up and was formally diagnosed with accidental tattooing of the tympanic membrane secondary to trauma. Despite a previous history of bilateral middle ear effusion, she had fully recovered and experienced no other major medical events. The ENT physician captured an image of the tympanic membrane (Figure 4). Given the absence of current symptoms or otolaryngologic complications, she was instructed to follow up every three months for monitoring.

## Discussion

The tympanic membrane is a key component of the auditory system. Injuries to it can result in perforation, discoloration, conductive hearing loss, infection, barotrauma, or foreign body insertion. Although rare, clues to diagnosing tympanic membrane discolorations may include hemorrhages, foreign bodies, infections, and anatomical abnormalities.

Upon reviewing the literature, there have been very few cases describing abnormal pigmentation on the tympanic membrane. For example, one case involved a young man with a white mass in his middle ear cavity, initially misdiagnosed as middle ear cholesteatoma [3]. However, further studies revealed an anatomical abnormality: a high-riding jugular bulb covered in abnormally thick bone [3]. Another case described symmetrical bilateral black dots on the tympanic membranes of an elderly man, but no specific diagnosis was identified [4]. A similar case of unilateral dark pigmentation was eventually attributed to an autosomal recessive condition called alkaptonuria, which the patient in our case did not have [5].

Despite the variety of documented cases, none describe a similar persistent unilateral black pigmentation as seen in this patient. This led to a prolonged period without a definitive clinical diagnosis. Typically, nonspecific dark markings on the tympanic membrane are attributed to the hemotympanum, a collection of blood in the middle ear cavity resulting in ecchymosis, usually from blunt trauma [6]. Although the hemotympanum resembles this patient’s presentation, it typically resolves with reduced inflammation and blood reabsorption, without scar formation. Sports-related injuries, which can cause tympanic membrane perforations and complications like infections or ossicular discontinuities [7], were also inconsistent with this case.

In this case, the initial consideration was residual graphite from the pencil that may have remained on the tympanic membrane. The absence of granulation tissue and foreign body reactions made graphite tattooing a strong possibility, as graphite is inert and nonirritating, supporting the minimal inflammatory response observed. It is important to note that individual reactions to foreign bodies can vary, and any foreign body in the ear poses a risk of infection and other complications. Fungal and bacterial infections were ruled out, as the patient did not show typical inflammatory responses. Additionally, there was concern about a potential hematoma, tympanosclerosis, or melanocytic nevus or melanoma, prompting a CT scan. The CT findings supported the diagnosis of accidental tattooing of the tympanic membrane secondary to trauma.

## Conclusions

Tympanic membrane injuries and discolorations pose diagnostic challenges due to their rarity and diverse etiologies. This case underscores the importance of thorough clinical examination, imaging, and continuous learning in otolaryngology. It was determined that the most likely diagnosis was residual graphite from pencil trauma. Detailed investigations and imaging helped refine the diagnosis and improve patient outcomes. This case highlights the need for heightened awareness of such rare presentations to ensure timely and appropriate specialist referrals.
